# 

**Published:** 1897-12

**Authors:** 


### CONTENTS:

### SELECTIONS.

Article I.—Insensibility to Pain.... 337
" II.—The Entrance Examinations in Dental Colleges....... 339
" III.—The Possibilities of Prosthetic Dentistry. By E. J. Perry, D. D. S.... 343
" IV.—Treatment of Abscess. By Dr. Monroe.... 349
" V.—The Century's Progress in Chemistry. By Henry Smith Williams, M. D.... 354
" VI.—Trichloracetic Acid as a Solvent of Necrosis. By H. W. Allwine, D. D. S.... 362
" VII.—Children's Teeth as Indicators of Disturbed Nutrition. By S. Henry Dessau, M. D.... 363
" VIII.—The Dentist. By Dr. B. F. Sheppard.... 368
" IX.—Practical Things in Dental Practice. By J. G. Templeton, A. M., D. D. S.... 370

### OBITUARY.

Dr. DeWilton Snowden Dead.... 374
Dr. Evans.... 376

### MONTHLY SUMMARY.

Faulty Nutrition.... 378

A Colored Lady Doctor.... 379

For Inflamed Eyes.... 379

A Good Cement.... 379

A Lake of Petroleum.... 380

Prevention of Tartar.... 380

What to Try.... 381

English People's Teeth.... 381

The Micromotoscope.... 382

Popular Errors Concerning Snakes.... 383

Removal of Blood Stains.... 383

Preserving Gutta-Percha.... 384

Massage of the Gums.... 384

To Cement Metal to Glass.... 384

